# Antiplatelet therapy trends in Chinese ischemic stroke patients 2019–2024

**DOI:** 10.1038/s41598-025-22390-8

**Published:** 2025-11-05

**Authors:** Mingfen Wu, Hailun Jiang, Aning Sun, Bin Zhu, Zhigang Zhao

**Affiliations:** Department of Pharmacy, Beijing Tiantan Hospital, Capital Medical University, No. 119 South Fourth Ring West Road, Fengtai District, Beijing, 100070 People’s Republic of China

**Keywords:** Ischemic stroke, Antiplatelet drugs, Utilization status, Prescription trends, Health care, Neurology

## Abstract

To analyze the current status and trends of antiplatelet medication use among ischemic stroke (IS) patients in China. This cross-sectional study utilized data from the China Hospital Prescription Analysis Database (2019–2024), analyzing 1,505,850 prescriptions. Mann–Kendall tests were employed to analyze the trends of prescriptions and cost, while log-linear models assessed changes in medication proportions. Defined daily cost (DDC) was calculated to evaluate economic differences. Among 1,505,850 prescriptions analyzed, 63.5% were for male patients, and 64.6% were for patients aged 65 and older. The patients median age of the prescriptions were 69 years (IQR: 61–78).Total antiplatelet prescriptions increased significantly from 300,573 in 2019 to 336,142 in 2024 (*P* = 0.024). Aspirin remained predominant (52.7%), while clopidogrel use declined significantly (46.6% to 40.9%, *P* = 0.002). Ticagrelor and indobufen showed rapid growth (both *P* < 0.001). Significant economic variations were observed: aspirin had the lowest DDC (¥0.57/DDD), while clopidogrel (¥3.60/DDD) and ticagrelor (¥6.61/DDD) decreased post-national centralized volume-based procurement policy (*P* < 0.001). Regional disparities were notable, with Zhengzhou (*P* < 0.001) and Hangzhou (*P* = 0.008) showing the fastest growth, while Tianjin (*P* < 0.001) and Shanghai (*P* = 0.003) declined significantly. Cilostazol demonstrated 104% regional DDC variation (Shenyang ¥16.96/DDD vs. Harbin ¥8.29/DDD). Antiplatelet prescription counts in Chinese IS patients increased in the past five years, with aspirin remaining the most widely used and cost-effective option. The treatment landscape shows “traditional drug dominance with new drug growth”, marked by significant economic and regional variations, necessitating guideline-aligned and policy-informed optimization of medication strategies.

## Introduction

Ischemic stroke (IS) remains a leading cause of mortality and disability worldwide, with antiplatelet therapy serving as a cornerstone in secondary prevention strategies to reduce recurrence risk. The 2023 Chinese guideline^1^, alongside current international standards from the American Heart Association/American Stroke Association (AHA/ASA)^2^ and the European Stroke Organisation (ESO)^3^, consistently endorses aspirin and clopidogrel as first-line pharmacotherapies. However, emerging antiplatelet agents such as ticagrelor^4,5^ and indobufen^6,7^ have demonstrated accumulating evidence and expanding clinical applications. Notwithstanding these advancements, systematic evaluations of actual antiplatelet utilization patterns, regional disparities, and associated economic burdens among Chinese IS patients remain lacking. Leveraging a nationwide multicenter prescription database, this study comprehensively delineates prescription trends, cost variations, and geographical heterogeneity in antiplatelet drug use for IS patients. The findings provide robust empirical evidence to optimize clinical medication practices, inform health insurance policy development, and guide updates to clinical practice guidelines.

## Materials and methods

### Study design and ethics

This retrospective observational study employed prescription data to analyze patterns of antiplatelet utilization in clinical practice. The study protocol was approved by the Ethics Committee of Beijing Tiantan Hospital, Capital Medical University (Approval No. KY2025-031-01). Due to the retrospective nature of the study, the Ethics Committee of Beijing Tiantan Hospital, Capital Medical University waived the need of obtaining informed consent. This study complies with the principles of the Declaration of Helsinki and the Regulations on Ethical Review of Biomedical Research Involving Human Subjects in China. All participating hospitals provided de-identified data in compliance with China’s national privacy regulations and institutional requirements.

### Data collection

Prescription data were sourced from the China Hospital Prescription Analysis Cooperation Program, encompassing 150 medical institutions across 9 major regions, including Beijing, Shanghai, Guangzhou, Tianjin, Zhengzhou, Chengdu, Harbin, Shenyang and Hangzhou. This database has been extensively utilized in pharmacoepidemiological research, employing a systematic sampling method to annually extract 40 days of prescriptions (two non-consecutive weekdays per quarter) from participating hospitals, followed by de-identification to ensure patient confidentiality. The dataset encompasses structured variables including geographic location (city), prescription identification codes, patient demographics (gender, age), clinical departments, diagnoses, medication details (e.g., drug names, dosages), and associated costs.

#### Inclusion criteria

(1) Diagnosed with IS; (2) Age ≥ 18 years; (3) Prescriptions issued between 2019 and 2024; (4) At least one antiplatelet medication prescribed (aspirin, aluminum magnesium aspirin, clopidogrel, ticagrelor, cilostazol, indobufen, dipyridamole).

#### Exclusion criteria

Prescriptions with missing critical information.

### Data analysis

At data extraction (November 2024), the database contained records only through Q2 2024. As full-year 2024 data were unavailable, we extrapolated the annual sampled prescription volume by doubling the aggregate Q1–Q2 2024 count, assuming constant prescription patterns throughout the year. This derived estimate was used in subsequent trend analyses.

The annual antiplatelet medication costs for IS patients were calculated as the sum of all antiplatelet drug expenditures within the year. Costs are expressed in Chinese Yuan (¥) per Defined Daily Dose (DDD), with two decimal places. As 2024 data were only available for the first two quarters, annual costs were estimated by doubling the observed upper half-year costs. We employed the Defined Daily Dose Cost (DDC) to evaluate economic efficiency across medications, which quantifies the financial burden per standardized daily dose. A higher DDC indicates greater medication expense and patient economic burden. The formula is defined as^8^:

$$DDC = \frac{{Total Drug {\text{Cos}} t}}{Total Drug Consumption/DDD}\;\left( {Note: \, DDD \, values \, adhere \, to \, WHO \, standardized \, criteria} \right)$$

Data extraction and preprocessing were conducted using Microsoft Access. To analyze temporal trends in prescription volumes and medication costs, the non-parametric Mann–Kendall test was employed; percentage trends were evaluated via log-linear regression modeling. All statistical analyses and visualizations were performed using SPSS 26.0 and GraphPad Prism 8.0.2 (GraphPad Software, San Diego, CA). Statistical significance was defined as *P* < 0.05.

## Results

### Basic characteristics of the prescriptions

A total of 1,505,850 prescriptions for antiplatelet medications were included in this study. Aspirin accounted for the highest proportion of prescriptions (792,865 prescriptions, 52.7%), followed by clopidogrel (648,053 prescriptions, 43.0%). In contrast, cilostazol (28,444 prescriptions, 1.9%), indobufen (18,048 prescriptions, 1.2%), ticagrelor (17,863 prescriptions, 1.2%), and dipyridamole (577 prescriptions, 0.04%) were less frequently prescribed.

63.5% of the prescriptions were for male patients, and 64.6% of the prescriptions were for older adults. The patients median age of the prescriptions were 69 years (IQR: 61–78). Regional distribution showed that prescriptions originated predominantly from Shanghai (15.9%), followed by Beijing (14.9%) and Guangzhou (14.9%). Regarding patient sources, 60.1% of prescriptions were for inpatients, and 87.5% of drug costs were reimbursed by medical insurance. Departmental distribution indicated that neurology departments accounted for 61.1% of prescriptions, whereas emergency departments (2.5%) and neurosurgery departments (2.3%) represented smaller proportions. Cost analysis demonstrated significant variability across medications, with indobufen exhibiting the highest median drug cost (¥194.74) and dipyridamole the lowest (¥12.00). Detailed data are presented in Table 1.

**Table 1:** Demographic characteristics of antiplatelet prescriptions for IS patients in China, 2019–2024.

Characteristics	Total	Antiplatelet drugs					
		Aspirin	Clopidogrel	Ticagrelor	Cilostazol	Indobufen	Dipyridamole
Total, n (%)	1,505,850 (100)	792,865 (100)	648,053 (100)	17,863 (100)	28,444 (100)	18,048 (100)	577 (100)
Gender, n (%)							
Male	955,996 (63.5)	506,641 (63.9)	406,977 (62.8)	12,575 (70.4)	18,682 (65.7)	10,779 (59.7)	342 (59.3)
Female	549,854 (36.5)	286,224 (36.1)	241,076 (37.2)	5288 (29.6)	9762 (34.3)	7269 (40.3)	235 (40.7)
Age [median (IQR)]	69 (61–78)	68 (60–76)	70 (62–80)	67 (59–74)	72 (64–84)	70 (62–79)	71 (61–80)
18–64, n (%)	532,459 (35.4)	300,495 (37.9)	211,265 (32.6)	7342 (41.1)	7481 (26.3)	5685 (31.5)	191 (33.1)
≥ 65, n (%)	973,391 (64.6)	492,370 (62.1)	436,788 (67.4)	10,521 (58.9)	20,963 (73.7)	12,363 (68.5)	386 (66.9)
City, n (%)							
Beijing	225,060 (14.9)	125,807 (15.9)	90,416 (13.9)	3530 (19.8)	1261 (4.4)	4034 (22.4)	12 (2.1)
Shenyang	209,846 (13.9)	129,650 (16.3)	72,833 (11.2)	1294 (7.2)	3794 (13.3)	2265 (12.5)	10 (1.7)
Chengdu	153,975 (10.2)	85,500 (10.8)	63,680 (9.8)	1097 (6.1)	1920 (6.8)	1585 (8.8)	193 (33.4)
Guangzhou	224,794 (14.9)	104,577 (13.2)	107,450 (16.6)	3490 (19.5)	6887 (24.2)	2135 (11.8)	255 (44.2)
Harbin	71,096 (4.7)	38,490 (4.9)	29,681 (4.6)	1498 (8.4)	18 (0.1)	1409 (7.8)	0 (0)
Hangzhou	175,751 (11.7)	92,118 (11.6)	78,476 (12.1)	1561 (8.7)	2795 (9.8)	755 (4.2)	46 (8.0)
Shanghai	240,167 (15.9)	127,233 (16.0)	101,002 (15.6)	1831 (10.3)	8552 (30.1)	1549 (8.6)	0 (0)
Tianjin	127,699 (8.5)	45,252 (5.7)	76,289 (11.8)	457 (2.6)	3144 (11.0)	2557 (14.2)	0 (0)
Zhengzhou	77,462 (5.1)	44,238 (5.6)	28,226 (4.4)	3105 (17.4)	73 (0.3)	1759 (9.7)	61 (10.6)
Patient source							
Inpatient	905,617 (60.1)	461,604 (58.2)	407,847 (62.9)	13,612 (76.2)	14,246 (50.1)	8114 (45.0)	194 (33.6)
Outpatient	600,233 (39.9)	331,261 (41.8)	240,206 (37.1)	4251 (23.8)	14,198 (49.9)	9934 (55.0)	383 (66.4)
Reimbursement type							
Full or partial	1,317,610 (87.5)	689,477 (87.0)	572,632 (88.4)	13,816 (77.4)	24,911 (87.6)	16,336 (90.5)	438 (75.9)
Self-funded	182,870 (12.1)	100,065 (12.6)	73,634 (11.3)	3954 (22.1)	3511 (12.3)	1568 (8.7)	138 (23.9)
Other types	5370 (0.4)	3323 (0.4)	1787 (0.3)	93 (0.5)	22 (0.1)	144 (0.8)	1 (0.2)
Departments of care							
Neurology	920,163 (61.1)	482,856 (60.9)	403,522 (62.3)	6114 (34.2)	17,143 (60.3)	10,376 (57.5)	152 (26.3)
Neurosurgery	34,392 (2.3)	18,915 (2.4)	13,760 (2.1)	1262 (7.1)	292 (1.0)	121 (0. 7)	42 (7.3)
Emergency	37,388 (2.5)	21,523 (2.7)	15,166 (2.3)	377 (2.1)	148 (0.5)	173 (0.9)	1 (0.2)
Other	513,907 (34.1)	269,571 (34.0)	215,605 (33.3)	10,110 (56.6)	10,861 (38.2)	7378 (40.9)	382 (66.2)
Cost, CNY [median (IQR)]	15.00 (2.00–20.00)	15.00 (0.52–15.30)	14.70 (2.54–68.32)	16.90 (3.86–81.76)	43.20 (8.58–231.00)	194.74 (27.96–395.36)	12.00 (1.17–20.00)

IQR, interquartile range; CNY, Chinese Yuan (¥).

### Prescription trends of antiplatelet medications

**Fig. 1:**
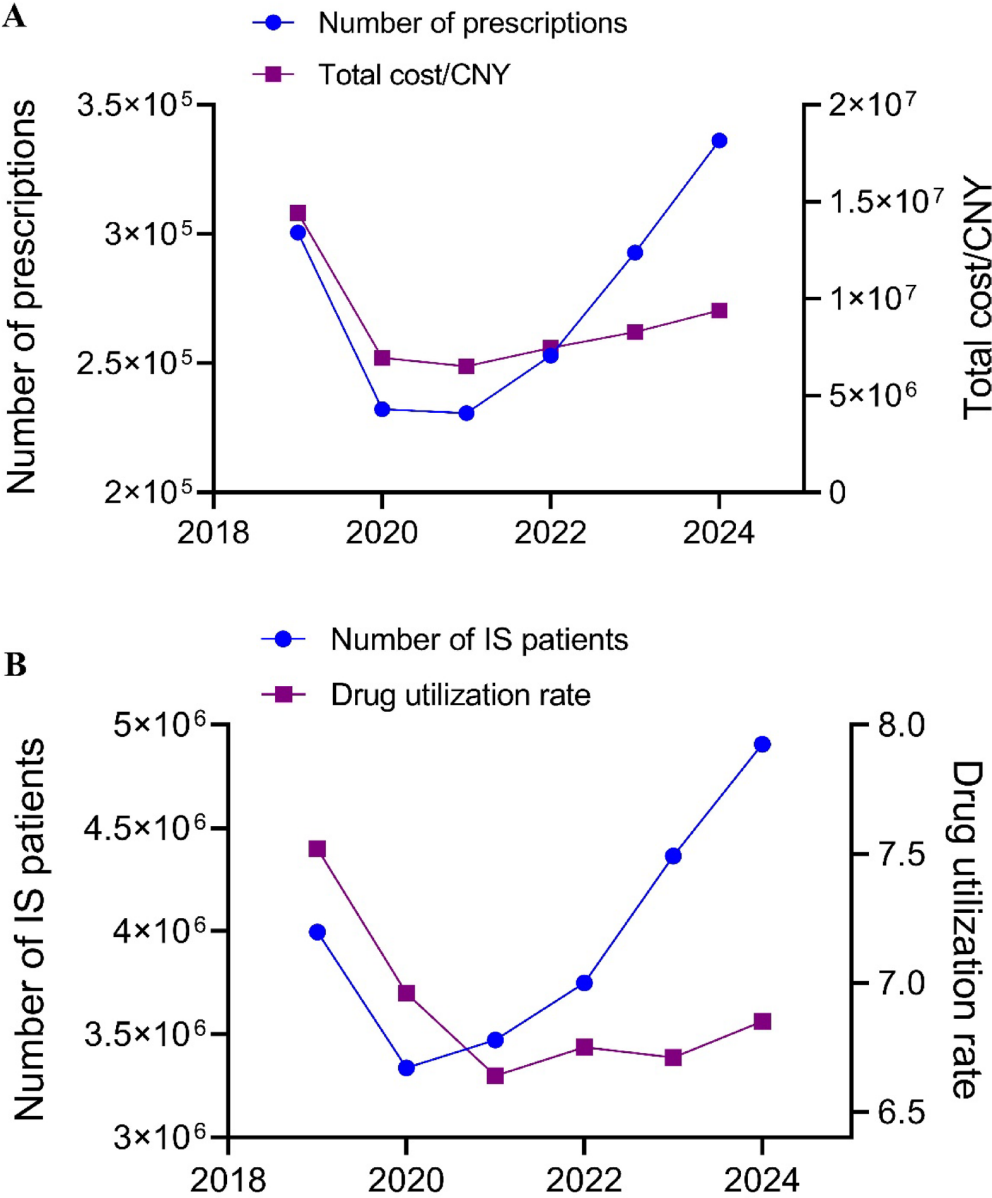
The trends of antiplatelet prescriptions and IS patients in China from 2019 to 2024 (2024 data annualized). (**A**) Annual number of prescriptions and cost; (**B**) Annual number of patients and antiplatelet medication utilization rate.

Table 2 illustrates the number and proportion of antiplatelet prescriptions for IS patients in China from 2019 to 2024, and Fig. 1 displays the trends of antiplatelet prescriptions and IS patients. As shown in Table 2 and Fig. 1A, the annual total prescription counts demonstrated a significant increase from 300,573 in 2019 to 336,142 (estimated value) in 2024 (P_1_ = 0.024). Among individual agents, aspirin was maintained dominance, with its prescription proportion fluctuating between 50.6% and 60.5%. Despite a nadir of 52.3% in 2022, overall prescriptions for aspirin showed a significant upward trend (P_1_ = 0.038), underscoring its clinical primacy as a first-line antiplatelet agent. Notably, clopidogrel exhibited a marked decline, with its prescription proportion decreasing from 46.6% in 2019 to 40.9% in 2024 (P_1_ = 0.002). In contrast, ticagrelor displayed the most pronounced growth (P_1_ < 0.001, P_2_ < 0.001), reaching a 1.9% prescription proportion in 2024, reflecting accelerated clinical adoption of novel P2Y_12_ receptor inhibitors. Particularly striking was the rapid surge in indobufen use (P_1_ < 0.001, P_2_ < 0.001), with its prescription proportion rising from 0.2% in 2019 to 2.7% in 2024. Cilostazol usage remained relatively stable (P_1_ = 0.214, P_2_ = 0.412), whereas dipyridamole retained an extremely low utilization rate with no significant temporal trend (P_1_ = 0.721, P_2_ = 1.000), indicating limited clinical application.

**Table 2:** Number and proportion of antiplatelet prescriptions in IS patients (2019–2024).

Drug name	2019	2020	2021	2022	2023	2024*	P_1_	P_2_
Aspirin	151,987 (50.6)	126,749 (54.6)	139,624 (60.5)	132,411 (52.3)	153,905 (52.6)	88,189 (52.5)	0.038	0.186
Clopidogrel	140,158 (46.6)	97,974 (42.2)	81,602 (35.4)	109,157 (43.2)	121,851 (41.6)	68,816 (40.9)	0.002	0.002
Ticagrelor	2264 (0.8)	2061 (0.9)	2651 (1.1)	3079 (1.2)	4579 (1.6)	3229 (1.9)	< 0.001	< 0.001
Cilostazol	5524 (1.8)	4113 (1.8)	4572 (2.0)	4901 (1.9)	6000 (2.0)	3334 (2.0)	0.214	0.412
Indobufen	507 (0.2)	1184 (0.5)	2121 (0.9)	3333 (1.3)	6434 (2.2)	4469 (2.7)	< 0.001	< 0.001
Dipyridamole	133 (0.0)	112 (0.0)	133 (0.1)	84 (0.1)	81 (0.0)	34 (0.0)	0.721	1.000
Total	300,573 (100)	232,193 (100)	230,703 (100)	252,965 (100)	292,850 (100)	168,071 (100)	0.024	–

Prescription counts and utilization rates are expressed as n (%). P₁: Mann–Kendall test for annual prescription count trend. P₂: Log-linear test for proportion trend. *The 2024 data represent the amount for the first and second quarters (half-year), and the trend calculation was estimated based on the annualized amount (× 2).

To account for variations in patient population size, we analyzed trends in the number of IS patients and antiplatelet medication utilization rates (Fig. 1B). While the number of IS patients temporarily declined to 3,337,179 in 2020, it rebounded to 4,906,204 by 2024, a 22.8% increase compared to 2019. Concurrently, antiplatelet utilization rates exhibited a “decline-then-recovery” pattern, decreasing from 7.5% in 2019 to a trough of 6.6% in 2021 before gradually rising to 6.9% in 2024.

### Trend analysis of antiplatelet medications cost

Table 3 illustrates trends in antiplatelet medication costs for IS patients from 2019 to 2024. Total antiplatelet costs demonstrated a significant downward trend, decreasing from ¥14,430,357.49 in 2019 to ¥8,279,138.10 in 2023, with a half-year estimate of ¥4,699,641.21 in 2024 (annualized to ¥9,399,282.42; *P* = 0.024; Fig. 1A). Notably, cost proportion of aspirin increased from 11.4% to 21.7% (*P* = 0.002), reflecting its consolidating clinical role. The cost clopidogrel experienced a significant reduction (*P* = 0.024), with its proportion dropping sharply from 82.1% to 41.1% (*P* < 0.001). Indobufen showed the most striking surge, with costs increasing markedly (*P* = 0.002) and its proportion skyrocketing from 1.0% to 24.2% (*P* < 0.001). Cilostazol demonstrated steady growth (*P* = 0.024), with its cost proportion rising from 3.8% to 10.2% (*P* = 0.008). Ticagrelor exhibited no significant changes in costs or proportional share (*P* > 0.05).

**Table 3:** Trends in antiplatelet medication prescription costs for IS patients (2019 ~ 2024, CNY).

Drug name	2019	2020	2021	2022	2023	2024*	P_1_	P_2_
Aspirin	1,649,076.61 (11.4)	1,584,458.93 (22.8)	1,209,153.41 (18.6)	1,546,235.66 (20.7)	1,738,648.74 (21.0)	1,019,080.46 (21.7)	0.463	0.002
Clopidogrel	11,842,921.42 (82.1)	4,192,234.22 (60.4)	3,941,697.85 (60.5)	4,037,942.92 (54.1)	3,640,505.84 (44.0)	1,932,057.43 (41.1)	0.024	< 0.001
Ticagrelor	245,228.73 (1.7)	270,062.77 (3.9)	144,600.59 (2.2)	156,143.11 (2.1)	204,791.47 (2.5)	130,475.43 (2.8)	0.345	0.186
Cilostazol	549,417.05 (3.8)	497,451.46 (7.2)	589,403.32 (9.0)	666,102.37 (8.9)	823,592.60 (9.9)	479,453.63 (10.2)	0.024	0.008
Indobufen	141,370.28 (1.0)	390,324.86 (5.6)	628,896.34 (9.7)	1,054,742.44 (14.1)	1,870,804.51 (22.6)	1,138,174.15 (24.2)	0.002	< 0.001
Dipyridamole	2343.40 (0.0)	1757.05 (0.1)	1907.30 (0.0)	1380.35 (0.0)	794.94 (0.0)	400.11 (0.0)	0.024	0.721
Total cost	14,430,357.49 (100)	6,936,289.29 (100)	6,515,658.81 (100)	7,462,546.85 (100)	8,279,138.10 (100)	4,699,641.21 (100)	0.024	0.002

Data are presented as amount (%); CNY, Chinese Yuan. P₁: Mann–Kendall test for annual cost trend. P₂: Log-linear test for proportion trend. *The 2024 data represent the amount for the first and second quarters (half-year), and the trend calculation was estimated based on the annualized amount (× 2).

### Cost evaluation using DDC

This study employed DDC as a standardized metric to systematically compare the economic efficiency of different antiplatelet medications (Table 4). The analysis revealed significant gradients in DDC values across drugs. Aspirin demonstrated optimal cost-effectiveness (¥0.57/DDD). Clopidogrel (¥3.60/DDD) and ticagrelor (¥6.61/DDD) cost 6.32-fold and 11.60-fold higher than aspirin, respectively. Cilostazol (¥12.45/DDD) and indobufen (¥14.11/DDD) incurred the highest treatment costs, representing 21.84 times (¥11.88/DDD absolute difference) and 24.75 times (¥13.54/DDD absolute difference) the cost of aspirin, respectively. Notably, dipyridamole, while demonstrating a moderate DDC (¥2.07/DDD), showed substantially lower total consumption and expenditure compared to other agents. Log-linear regression analysis indicated remarkable cost reductions for clopidogrel and ticagrelor (*P* < 0.001), potentially attributable to China’s national centralized volume-based procurement (NCVBP) policy. In contrast, traditional agents (aspirin, dipyridamole) and novel mechanism drugs (cilostazol, indobufen) maintained relatively stable pricing systems.

**Table 4:** DDC of different antiplatelet medications.

Drug name	Total cost/CNY	Total drug consumption	DDD	DDDs	DDC/CNY
Aspirin	9,062,940.16	15,989,427 pill	1 pill	15,989,427.00	0.57
Clopidogre	29,523,958.04	614,376,025 mg	75 mg	8,191,680.33	3.60
Ticagrelor	1,151,302.10	31,360,590 mg	180 mg	174,225.50	6.61
Cilostazol	3,605,420.43	57,906,200 mg	200 mg	289,531.00	12.45
Indobufen	5,224,312.58	74,039,000 mg	200 mg	370,195.00	14.11
Dipyridamole	8583.15	1,662,325 mg	400 mg	4155.81	2.07

DDD (Defined Daily Dose): WHO-standardized average maintenance dose for adults. DDDs (Defined Daily Doses): Total drug consumption divided by DDD; DDDs = Total Consumption (mg)/DDD (mg/day); DDC (Defined Daily Cost): The average cost per DDD; DDC = Total Cost/DDDs. CNY, Chinese Yuan (¥).

As shown in Table 5, DDC trends diverged significantly across drug classes. Clopidogrel and ticagrelor exhibited statistically significant DDC declines (*P* < 0.001), aligning with the implementation timeline of China’s NCVBP policy. Aspirin maintained stable DDC values (*P* > 0.05). Dipyridamole showed minor, non-significant relative reductions (*P* = 0.062), while cilostazol and indobufen displayed minimal fluctuations (*P* > 0.05). These findings suggest heterogeneous pricing trends across antiplatelet classes, likely influenced by drug-specific characteristics, market competition dynamics, and health insurance policies.

**Table 5:** Trends in DDC of different antiplatelet medications (2019–2024).

Drug name	2019	2020	2021	2022	2023	2024	*P*
Aspirin	0.60	0.60	0.55	0.54	0.55	0.54	0.218
Clopidogrel	8.31	3.34	2.77	2.63	2.19	2.10	< 0.001
Ticagrelor	16.91	16.07	5.84	4.55	4.16	3.76	< 0.001
Cilostazol	13.11	12.98	13.19	12.16	11.70	12.16	0.736
Indobufen	14.93	14.38	14.35	14.19	14.07	13.80	0.152
Dipyridamole	2.26	2.09	2.05	1.87	1.97	1.91	0.062

*P*, *P*-value from log-linear regression analysis for DDC trend. DDC values in CNY.

### Regional analysis of antiplatelet medication utilization

**Fig. 2:**
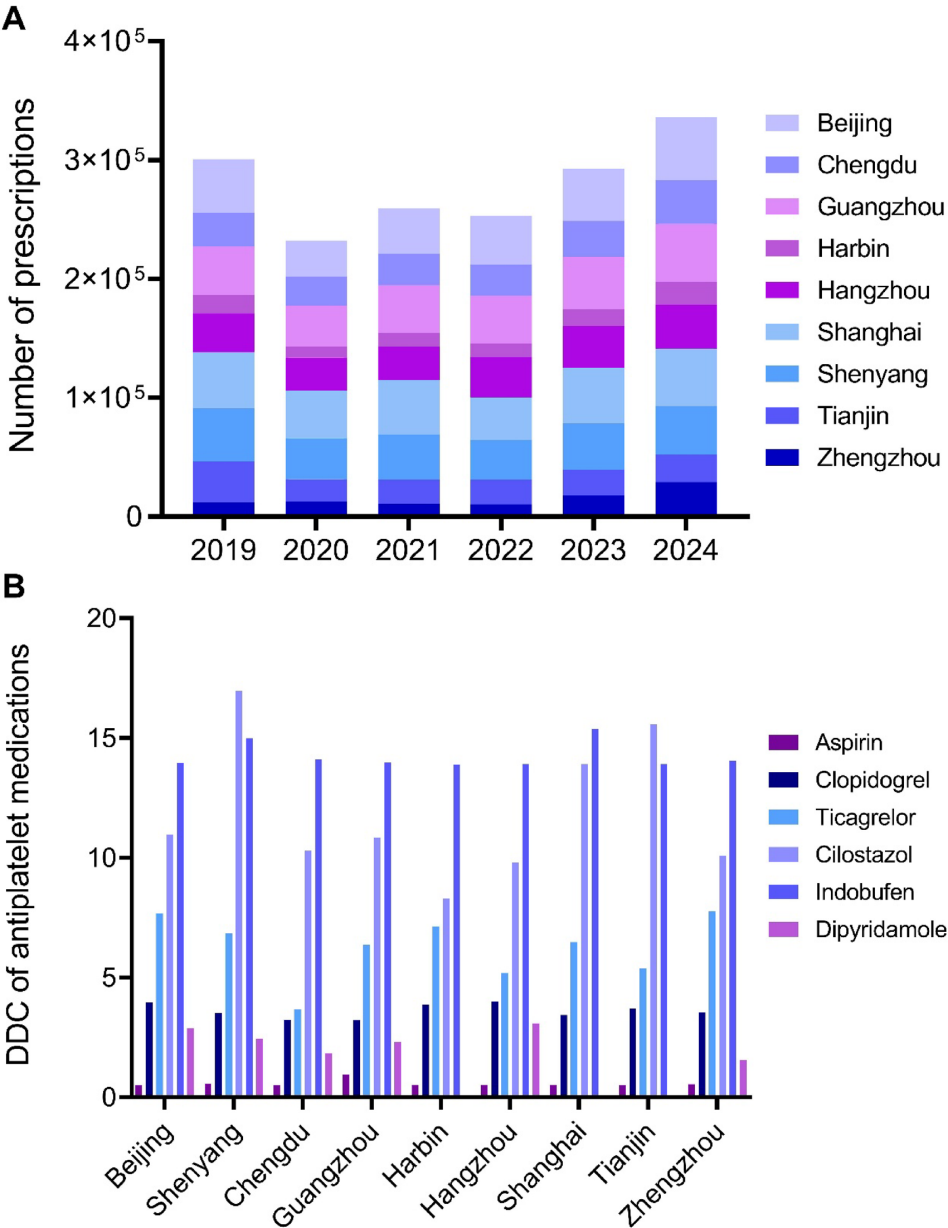
Trends in antiplatelet prescriptions across 9 cities (2024 data annualized) (**A**) Number of prescriptions; (**B**) DDC.

Figure 2 displays the trends in antiplatelet prescriptions across 9 cities from 2019 to 2024. The dynamic trends in the number of antiplatelet prescriptions are illustrated in Fig. 2A. Table 6 further delineates significant regional disparities through two dimensions: prescription counts and annual proportion trends. Regarding prescription count trends, seven cities (Beijing, Chengdu, Guangzhou, Harbin, Hangzhou, Shanghai, Zhengzhou) exhibited significant growth, with Zhengzhou (*P* = 0.002) and Hangzhou (*P* = 0.004) showing the most pronounced growth. Guangzhou maintained stable growth (*P* = 0.009), while Shenyang (*P* = 0.31) and Tianjin (*P* = 0.19) displayed no statistically significant changes. For annual prescription proportion trends, Zhengzhou (*P* < 0.001) and Hangzhou (*P* = 0.008) led in proportional growth, followed by Harbin (*P* = 0.012). However, Tianjin (*P* < 0.001), Shanghai (*P* = 0.003), and Shenyang (*P* = 0.033) experienced significant reductions. Chengdu and Guangzhou showed no significant proportional changes (*P* > 0.05). Regional disparities highlight that Hangzhou and Zhengzhou demonstrated simultaneous increases in both prescription counts and proportions, suggesting escalating demand for antiplatelet therapies in these regions.

**Table 6:** Trends in the number and proportion of antiplatelet prescriptions by region (2019–2024).

Region	2019	2020	2021	2022	2023	2024	P_1_	P_2_
Beijing	44,881(14.9)	30,573 (13.2)	38,354 (14.8)	40,714 (16.1)	43,992 (15.0)	53,092 (15.8)	0.027	0.041
Chengdu	28,466 (9.5)	24,003 (10.3)	26,247 (10.1)	26,560 (10.5)	30,229 (10.3)	36,940 (11.0)	0.015	0.217
Guangzhou	40,907 (13.6)	34,693 (14.9)	40,227 (15.5)	40,058 (15.8)	44,608 (15.2)	48,602 (14.5)	0.009	0.538
Harbin	15,386 (5.1)	9241 (4.0)	11,524 (4.5)	11,526 (4.6)	13,803 (4.7)	19,232 (5.7)	0.042	0.012
Hangzhou	32,687 (10.9)	27,554 (11.9)	27,848 (10.7)	33,913 (13.4)	35,203 (12.0)	37,092 (11.0)	0.004	0.008
Shanghai	47,315 (15.8)	40,260 (17.4)	46,055 (17.8)	35,711 (14.1)	46,666 (16.0)	48,320 (14.4)	0.038	0.003
Shenyang	44,614 (14.8)	34,411 (14.8)	37,982 (14.7)	33,580 (13.3)	38,922 (13.3)	40,674 (12.1)	0.312	0.033
Tianjin	34,543 (11.5)	19,083 (8.2)	20,220 (7.8)	20,645 (8.2)	21,524 (7.4)	23,368 (6.9)	0.185	< 0.001
Zhengzhou	11,774 (3.9)	12,375 (5.3)	10,741 (4.1)	10,258 (4.0)	17,903 (6.1)	28,822 (8.6)	0.002	< 0.001

Prescription counts and proportions are expressed as n (%), where proportions represent the annual percentage of patients using antiplatelet medications. P₁: Mann–Kendall test for annual prescription count trend. P₂: Log-linear test for proportion trend. *The 2024 data represent the amount for the first and second quarters (half-year), and the trend calculation was estimated based on the annualized amount (× 2).

Figure 2B compares regional DDC values across cities. Key findings include: Aspirin maintained the lowest DDC (¥0.50–0.95/DDD) across all regions, confirming its cost-effectiveness as a first-line agent. Clopidogrel stabilized at ¥3.21–3.99/DDD (~ 6–eightfold higher than aspirin), representing mid-tier costs. Cilostazol and indobufen had the highest DDC values (¥8.29–16.96 and ¥13.90–15.38/DDD, respectively), indicating high economic burdens. Notably, cilostazol exhibited a 104% relative increase in DDC between regions (Shenyang: ¥16.96/DDD vs. Harbin: ¥8.29/DDD), representing an absolute difference of ¥8.67/DDD, potentially reflecting regional procurement policy or formulation preferences. The dosage of dipyridamole is relatively small, and the DDC value is 0 in some cities (Harbin, Shanghai, Tianjin), indicating discontinuation in these areas.

## Discussion

### Trends in antiplatelet medication utilization

This study reveals a discordant relationship between the growing IS patient population and declining antiplatelet medication utilization rates, a finding with critical clinical implications. Despite a relative 22.8% increase in IS patients (2019–2024; *P* = 0.024), antiplatelet utilization decreased from 7.5% to 6.9% during the same period. This discrepancy may reflect multifaceted challenges, including inconsistent implementation of clinical guidelines in primary care institutions^9,10^ and suboptimal adherence to secondary prevention regimens among IS patients^11^, highlighting the need for enhanced post-discharge follow-up protocols.

Current guidelines^1,12,13^ universally recommend aspirin (75–100 mg/day) or clopidogrel (75 mg/day) as first-line therapies for non-cardioembolic IS secondary prevention, while newer agents like ticagrelor and cilostazol are reserved for specific populations (e.g., CYP2C19 loss-of-function allele carriers^5^, aspirin-intolerant patients, or those at high gastrointestinal bleeding risk^14^). Consistent with these recommendations, our findings confirm that aspirin (52.7%) and clopidogrel (40.9%) remain cornerstone therapies. However, their utilization trends diverged significantly, aspirin maintained its dominant position but showed a fluctuating downward trajectory (*P* = 0.186), whereas clopidogrel experienced a sharper decline (46.6% to 40.9%; *P* = 0.002). The observed trends are likely driven by multifaceted determinants. First, there are emerging alternatives. The rapid rise of ticagrelor (*P* < 0.001) and indobufen (*P* < 0.001) reflects growing interest in potent P2Y_12_ inhibitors and novel antithrombotic mechanisms. Notably, ticagrelor’s combination with aspirin, supported by recent trials like THALES^15,16^, may be reshaping clinical practices, particularly in high-risk cohorts. Secondly, there are pharmacoeconomic factors. Despite price reductions for clopidogrel post-NCVBP policy (*P* < 0.001), utilization failed to increase proportionally. This suggests that cost reduction alone is insufficient to alter prescribing behaviors, necessitating complementary interventions such as guideline updates and physician education.

A striking anomaly lies in indobufen’s explosive growth (0.2% to 2.7%), far surpassing other newer agents. While China’s domestic guidelines endorse indobufen for its perceived lower bleeding risk, robust evidence from randomized controlled trials (RCTs) validating its efficacy and safety versus established therapies remains absent—a critical knowledge gap requiring prospective validation. Current real-world usage trends should therefore be interpreted with caution pending confirmatory RCT data. This warrants three priority actions. First, expand real-world studies to evaluate long-term clinical outcomes. Second, optimize clinical pathways to ensure rational use of novel agents. Third, strengthen pharmacovigilance systems to detect safety signals proactively. These findings underscore a dual imperative: adhere to guideline principles while dynamically integrating emerging evidence to balance innovation with cost-effectiveness, ultimately advancing personalized, precision stroke prevention.

Furthermore, population aging necessitates refined risk–benefit assessments in drug selection. This study found that 64.6% of antiplatelet prescriptions were for patients aged 65 and older. The rapidly growing use of ticagrelor and indobufen may reflect a clinical preference for safer thromboprophylactic options; however, the underrecognized risk of overusing agents lacking robust long-term outcome data warrants vigilant monitoring.

### Pharmacoeconomic analysis of antiplatelet therapies

This study provides a comprehensive evaluation of antiplatelet therapy costs in Chinese IS patients using the DDC metric, revealing three critical pharmacoeconomic characteristics. First, significant disparities in economic efficiency were observed across medications, with aspirin solidifying its role as a cost-effective cornerstone therapy (¥0.57/DDD), aligning with global guideline recommendations. Notably, China’s NCVBP policy drove statistically significant DDC reductions for clopidogrel (¥3.60 to ¥3.21/DDD, *P* < 0.001) and ticagrelor (¥6.61 to ¥5.88/DDD, *P* < 0.001), demonstrating tangible cost-saving effects of national healthcare reforms. However, high-cost agents like cilostazol (¥12.45/DDD) and indobufen (¥14.11/DDD) may limit accessibility, particularly in resource-constrained settings.

Comparative analysis with prior studies^17^ reveals enhanced contextual insights: while genotype-guided personalized regimens (e.g., cilostazol/ticagrelor substitution for clopidogrel) may optimize cost-effectiveness in select cohorts, aspirin and clopidogrel remain the most universally viable economic choices within China’s healthcare infrastructure. This underscores the necessity for multifaceted decision-making frameworks integrating stroke severity, regional resource availability, and real-world evidence. Furthermore, regional DDC disparities highlight systemic inequities—cilostazol’s 104% DDC variation between Shenyang (¥16.96/DDD) and Harbin (¥8.29/DDD)—suggesting divergent procurement policies and clinical preferences. The near-zero utilization of dipyridamole in cities like Shanghai and Tianjin aligns with its low guideline recommendation status and declining clinical demand.

To address these challenges, we propose a tri-level optimization strategy. First of all, it is the policy level. Prioritize cost-effective agents (e.g., aspirin/clopidogrel) in reimbursement frameworks; The second aspect is the regulatory level. Enhance monitoring of novel drug utilization (e.g., indobufen) through safety-efficacy-economic triage; Finally, there is the clinical aspect. Implement stratified treatment protocols balancing cost–benefit ratios. Future research should quantify cost-effectiveness thresholds to guide policy evolution and resource allocation.

### Potential drivers of regional disparities

Our findings revealed notable regional disparities in antiplatelet prescription trends between 2019 and 2024. Hangzhou, Zhengzhou, and Harbin exhibited concurrent significant growth in both the counts and proportion of prescriptions, whereas Tianjin, Shanghai, and Shenyang showed a marked decline in proportion.

Several factors may underlie these variations. The growth in emerging centers (e.g., Hangzhou, Zhengzhou, Harbin) likely reflects improved healthcare infrastructure, adherence to stroke guidelines, and the impact of recent national initiatives such as stroke center expansion and prevention campaigns. By contrast, more established markets (e.g., Tianjin, Shanghai, Shenyang) appear to have entered a plateau phase, where earlier policy gains have stabilized and prescribing is increasingly driven by precision medicine considerations, including genetic testing and bleeding risk stratification. This “precision and optimization” phase may result in stable or even declining prescription volumes despite continued adherence to evidence-based practice. Furthermore, regional procurement policies and local insurance formularies may influence prescribing behaviors, as reflected in the pronounced intercity variation in DDC values (e.g., cilostazol in Shenyang vs. Harbin).

Importantly, these regional disparities should not be interpreted as uniformly positive or negative. Rather, they represent distinct stages of stroke care standardization across China. Some regions remain in a stage of “popularization and expansion,” emphasizing basic adherence to guidelines, while others have transitioned toward “precision and optimization,” prioritizing individualized therapeutic strategies. Policy development must therefore be stage-specific: in emerging regions, efforts should prioritize strengthening infrastructure, expanding stroke center coverage, and enhancing physician training; in mature markets, policies should emphasize outcome monitoring, pharmacoeconomic evaluation, and rational use of high-cost agents. Such differentiated approaches will better align national stroke prevention goals with regional realities.

### Limitations and future directions

This study still has some limitations. First, data were predominantly sourced from secondary and tertiary hospitals, potentially underrepresenting prescribing patterns in primary care settings. Second, as a prescription-level analysis, we lacked clinical outcome metrics (e.g., stroke recurrence, bleeding events) and cost-effectiveness evaluations. Third, the extrapolation of the 2024 sampled prescription volume by doubling the Q1–Q2 data may introduce bias if prescribing patterns changed in the latter half of the year. Fourth, the long-term impacts of NCVBP policies require extended observation periods.

Future research should therefore adopt a multi-dimensional approach. First, real-world comparative effectiveness studies incorporating clinical outcomes are needed to clarify the impact of different antiplatelet regimens on stroke recurrence and safety. Second, region-specific investigations should evaluate how local healthcare infrastructure, insurance coverage, and patient-level characteristics shape prescribing behaviors, thereby informing differentiated policy interventions. Third, economic evaluations should quantify cost-effectiveness thresholds across regions to guide resource allocation. Lastly, longitudinal monitoring of novel agents such as indobufen is warranted to balance innovation, safety, and affordability.By integrating prescription data with clinical outcomes and regional health system characteristics, future studies can provide stronger evidence to support tailored, stage-specific strategies for optimizing antiplatelet therapy in ischemic stroke patients across China.

In conclusion, our prescription trend analysis systematically characterized two pivotal features of antiplatelet therapy in Chinese IS patients: (1) guideline-recommended agents (aspirin, clopidogrel) remained dominant, and (2) novel agents (ticagrelor, indobufen) exhibited rapid adoption growth. These findings provide critical evidence for refining secondary stroke prevention strategies. While adhering to established treatment protocols, clinicians should weigh the clinical value and cost-efficiency of innovative therapies. Policy interventions, particularly reimbursement frameworks, should guide resource allocation to advance precision and accessibility in stroke prevention.

## Data Availability

The datasets used and/or analysed during the current study available from the corresponding author on reasonable request.

## Abbreviations

IS: Ischemic stroke
DDC: Defined daily dose cost
DDD: Defined daily dose
NCVBP: National centralized volume-based procurement
WHO: World Health Organization
RCT: Randomized controlled trial
